# The World’s Fair—Accommodations

**Published:** 1893-04

**Authors:** 


### The World's Fair—Accommodations.

Much has been said pro and con in regard to the accommodation of visitors to the Fair and the expense involved. Undoubtedly if circumstances are favorable there will be the largest gatherings of people that have ever taken place on this continent. While there may be a disposition on the part of some of those whose business it will be to provide for the comfort of those who may attend, to extort, it can hardly be possible that the number of such will be very large. The people of Chicago cannot afford to permit anything of this kind to become general, nor to be practiced in any case with approval. The interests of the Fair and the city, and the good name of Chicago will be promoted by a fair, liberal and inviting policy.

The following statement from the authorities of the Fair seem to indicate that abundant provision will be made, and at reasonable rates for all who will attend:

"In answer to the many inquiries from boards of trade and similar bodies concerning the alleged plan to demand extortionate prices for accommodations in Chicago next summer, Major W. Marsh Kasson, at the request of the Director-General, has prepared the following report from the bureau of public comfort:

"This bureau has been established by the World's Columbian Exposition to co-operate, through its hotels and rooming departments, to the best of its ability, with the citizens of Chicago for the comfort and protection of visitors, to secure for them suitable and desirable lodging at fair and reasonable rates.

"The management is keenly alive to the fact that thousands of visitors will be deterred from visiting the city unless they can be fully assured on this point, hence every effort is being made to realize satisfactory results in this direction.

"Inquiries were sent out some time ago to householders having furnished rooms to let, to learn, as far as possible, prices that would be expected therefor, and the following statement gives the general average quotations received in reply, to cover accommodation for over 16,000 people in the best part of the city lying between North avenue and Seventy-ninth street.

"Price of rooms per day without board:

"Single room, single bed, one person, $1.35.

"Double room, double bed, one person, $2.12; two persons, $2.70.

"Double-bedded room, two double beds, two persons, $3.50.

"Double-bedded room, two double beds, three persons, $4.15.

"Double-bedded room, two double beds, four persons, $5.50.

"There certainly does not seem, according to these figures, to be any indication that citizens of Chicago will demand excessive rates for the accommodation of visitors to the Exposition, and they can be relied upon to sustain the good reputation of Chicago for fair and liberal treatment of its guests. One publishing house has a pamphlet now in press containing a list of over 10,000 places in the city of Chicago where furnished rooms can be had at moderate rates.

"The great mass of visitors will doubtless prefer the quiet and economy of furnished rooms such as alluded to, and apartment hotels, arranging to take their meals from day to day wherever it may suit their convenience. It is estimated that from 50,000 to 100,000 people can be served daily with meals within the Exposition grounds alone, while the number of hotels and restaurants is constantly increasing.

